# A Hybrid Convolutional and Recurrent Neural Network for Multi-Sensor Pile Damage Detection with Time Series

**DOI:** 10.3390/s24041190

**Published:** 2024-02-11

**Authors:** Juntao Wu, M. Hesham El Naggar, Kuihua Wang

**Affiliations:** 1 College of Civil Engineering and Architecture, Zhejiang University, Hangzhou 310058, China; 2 Geotechnical Research Centre, University of Western Ontario, London, ON N6A 5B9, Canada

**Keywords:** pile damage detection, multiple sensors, convolutional neural network, recurrent neural network, analytical solution

## Abstract

Machine learning (ML) algorithms are increasingly applied to structure health monitoring (SHM) problems. However, their application to pile damage detection (PDD) is hindered by the complexity of the problem. A novel multi-sensor pile damage detection (MSPDD) method is proposed in this paper to extend the application of ML algorithms in the automatic identification of PDD. The time-series signals collected by multiple sensors during the pile integrity test are first processed by the traveling wave decomposition (TWD) theory and are then input into a hybrid one-dimensional (1D) convolutional and recurrent neural network. The hybrid neural network can achieve the automatic multi-task identification of pile damage detection based on the time series of MSPDD results. Finally, the analytical solution-based sample set is utilized to evaluate the performance of the proposed hybrid model. The outputs of the multi-task learning framework can provide a detailed description of the actual pile quality and provide strong support for the classification of pile quality as well.

## 1. Introduction

Pile foundations play an important role in onshore and offshore engineering projects. Therefore, it is necessary to evaluate the integrity and quality of pile foundations and thus ensure the safety of supported superstructures. To detect the integrity of reinforced concrete (RC) piles, a variety of non-destructive testing techniques have been proposed successively, such as the low-strain pile integrity test (PIT) [1,2,3,4], sonic cross-hole logging (SCL) [5,6,7,8] and the parallel seismic (PS) method [9,10,11,12,13,14]. The existence of potential pile defects and unknown pile lengths can be detected by one method or a combination of multiple methods. Meanwhile, these techniques still have certain limitations, e.g., the PIT and lateral PIT [15,16] methods are convenient to implement, however the results may not be as reliable in the case of shallow and multiple defects, where the pile toe reflection is inevitably affected by the defect above. Similarly, the SCL test can be conducted along the entire length of the pile, but it only examines the area between the sonic logging tubes and cannot reflect the actual mechanical information of the pile cross-section. The PS results incorporate the whole mechanical information of both the pile and the surrounding soil, but the test requires significant preparatory work, which increases the cost. At the same time, most of these methods rely heavily on the operators’ experience, which is not conducive to automatic identification.

Machine learning (ML) algorithms are increasingly applied to structure health monitoring (SHM) [17,18,19,20]. Several researchers employed ML algorithms, such as support vector machine (SVM) and artificial neural network (ANN), to automatically recognize damage to steel truss [21,22], steel frames [23,24] and bridge structures [25,26,27]. Given the acceleration and velocity responses that are collected from the structure are time-series signals, there are two major ways to extract vibration-based features. The first method extracts statistical features from time-series signals and uses them as the input to ML algorithm frameworks [28,29,30]. The second method utilizes the one-dimensional (1D) convolutional neural network (CNN) to automatically extract the features [31,32,33,34]. The 1DCNN has the same advantages as the CNN and is capable of learning more comprehensive features (i.e., less susceptible to subjective experience). However, compared with superstructure monitoring, there are scarce studies on the application of ML algorithms to pile damage detection (PDD) due to the challenges of pile quality detection. Generally, existing pile detection techniques and their test results are not suitable for data-driven analysis (e.g., the test excitations of the PIT and PS methods are applied manually and are not repeatable; the reliability of one-sensor tests (PIT and lateral PIT) at the pile top is susceptible, and the PS test results with multiple sensors at desired depths are influenced by the structure above the sensors).

To overcome the limitations of existing pile detection techniques and further extend the application of ML algorithms in the automatic identification of PDD, a novel multi-sensor pile damage detection (MSPDD) method is proposed in this paper to evaluate the integrity of onshore and offshore pile foundations. Combined with the traveling wave decomposition (TWD) theory, the normalized MSPDD results can eliminate the vibration of the structure above the sensors. In order to handle the time-series MSPDD results, a hybrid one-dimensional (1D) convolutional and recurrent neural network is developed herein to automatically conduct multi-task pile damage detection. Finally, the analytical solution-based sample set is utilized to evaluate the performance of the proposed hybrid neural network.

## 2. Multi-Sensor Pile Damage Detection (MSPDD) Method

In order to overcome the limitations of existing pile quality detection methods and further integrate them with machine learning (ML) algorithms, a novel multi-sensor pile damage detection (MSPDD) method is proposed herein to evaluate the integrity of onshore and offshore pile foundations.

### 2.1. Operation Steps

In the MSPDD method, multiple (more than three) equally spaced sensors are installed along the pile shaft in the depth range of interest and are used to simultaneously receive the dynamic velocity responses induced by the test hammer strike. Different from the traditional pile integrity test (PIT) that utilizes only one sensor located at/near the pile top, employing multiple sensors at various depths gathers more information and is more flexible and reliable for complicated testing conditions. Combined with the raveling wave decomposition (TWD) theory [35,36] developed for equally spaced sensors, the MSPDD results can eliminate the effect of vibration of the structure above the sensors and thus reveal the integrity of the remainder below. Consequently, the MSPDD method and its test results are almost unaffected by shallow defects, enlarged pile tops and superstructures. Therefore, it can be utilized to evaluate piles with multiple defects and extremely long lengths.

**Figure 1:**
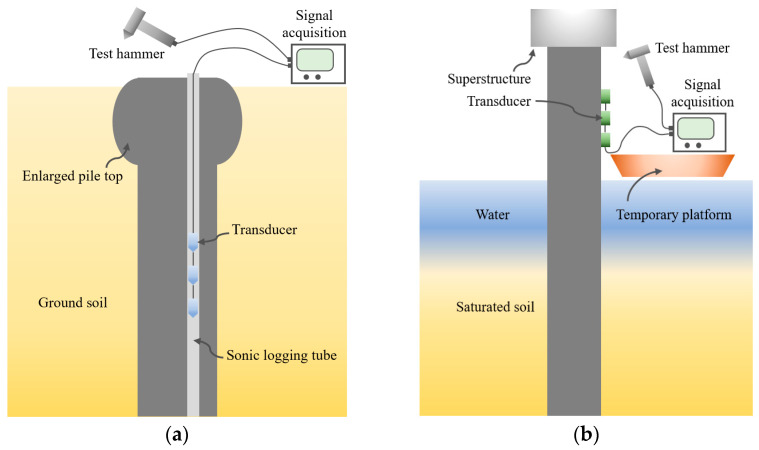
Layout of multiple sensors installed on (**a**) a pile with available sonic logging tubes; and (**b**) an extended pile shaft with superstructure.

To achieve the installation of multiple sensors at desired depths and make use of existing test techniques, some implementation ideas are proposed for onshore and offshore piles [37,38,39]. For example, as depicted in Figure 1a, if a reinforced concrete (RC) pile is qualified for the cross-hole sonic logging (CSL) test, a series of hydrophones (commonly used in the PS method) can be plugged into one of the water-filled sonic logging tubes located below the known defect (e.g., an enlarged pile top or a detected defect); Figure 1b demonstrates a series of transducers installed along the outer surface of an offshore pile to conduct the MSPDD test, where the sensors below the superstructure can remove the effects of the above structure after signal post-processing (as will be introduced in the ensuing section). For an RC pipe pile, the sensors can be installed on the pile’s inner wall. Even though the installation methods of sensors may be different considering various pile types and service environments, the multiple sensors are generally arranged at a specific depth with equal spacing to receive signals simultaneously. In addition, the receiving direction of the sensors should be consistent with the major direction of the hammer strike.

### 2.2. Signal Post-Processing

**Figure 2:**
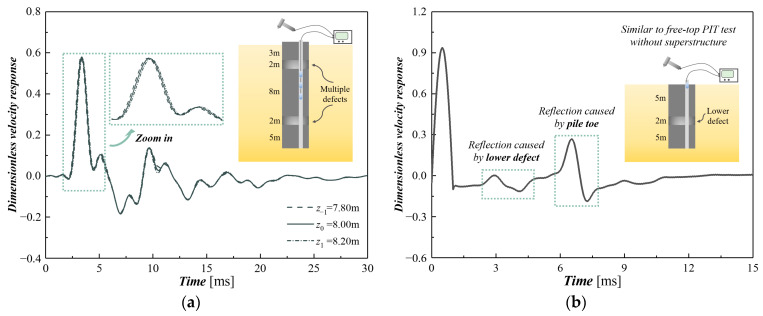
Post-processing for MSPDD method: (**a**) raw data collected by multiple sensors; (**b**) reconstructed MSPDD result employing traveling wave decomposition (TWD) theory.

After collecting a series of signals with a multi-channel data acquisition system (as demonstrated in Figure 2a), the traveling wave decomposition (TWD) theory is utilized to implement the post-processing. Considering three neighboring sensors as an example, the time-series signal can be expressed as the sum of downward- and upward-traveling waves, i.e.,

(1) $$v\left(z,t\right)=\xi \left(z-C_{p}t\right)+\eta \left(z+C_{p}t\right)$$

where *z* is the depth variable whose positive direction is downward; *t* is the time variable; *ξ*(⋅) and *η*(⋅) are the downward- and upward-traveling waveforms and *C_p_* denotes the one-dimensional traveling wave velocity of the RC pile (i.e., elastic wave velocity or shear wave propagating velocity corresponding to sensors collecting vertical or lateral components).

Since the effect of pile damping on the velocity amplitude of propagating waves is negligible due to the relatively small spacing between adjacent sensors (no more than 0.50 m in practice), the time-series signal collected by neighboring sensors satisfies the following relation:

(2) $$v\left(m,n\right)=\xi \left(m,n\right)+\eta \left(m,n\right)=\xi \left(z_{0}+m\Delta z,t+n\Delta t\right)+\eta \left(z_{0}+m\Delta z,t+n\Delta t\right)$$

where *m* = −1, 0, 1; *z*_0_ denotes the depth of the middle sensor; Δ*z* is the spacing between adjacent sensors; Δ*t* = Δ*z*/*C_p_*; *n* is a non-negative integer because of the causality of the linear time-invariant (LTI) system.

The traveling downward and upward waves yield the following:

(3) $$\left\{\begin{array}{l} \xi \left(m,n\right)=\xi \left(m-1,n-1\right)\\ \eta \left(m,n\right)=\eta \left(m+1,n-1\right) \end{array}\right.,n\geq 1$$

Comparing the velocities of the lower (*z*= *z*_0_ + Δ*z*) and upper (*z*= *z*_0_ − Δ*z*) sensors at *t* = *t* + Δ*t* with the velocity of the middle sensor (*z* = *z*_0_), respectively, we have

(4) $$\left\{\begin{array}{l} v\left(1,1\right)-v\left(0,0\right)=\eta \left(1,1\right)-\eta \left(0,0\right)=\eta \left(0,2\right)-\eta \left(0,0\right)\\ v\left(-1,1\right)-v\left(0,0\right)=\xi \left(-1,1\right)-\xi \left(0,0\right)=\xi \left(0,2\right)-\xi \left(0,0\right) \end{array}\right.$$

Since *v*(0,0), *v*(1,1) and *v*(−1,1) are known data collected by multiple sensors, the downward and upward waves passing through the middle sensor (i.e., *ξ*(0,0) and *η*(0,0)) can be solved by iterative calculation. By regarding the downward and upward waves as the input and output, the pseudo-frequency response (PFR) function for the structure below the sensors can be expressed as follows:

(5) $$H_{v}=\frac{\bar{\eta }\left(0,0\right)}{\bar{\xi }\left(0,0\right)}+1$$

where $\bar{\xi }$(0,0) and

$$\bar{\eta }$$

(0,0) are the Fourier transform (FT) of *ξ*(0,0) and *η*(0,0), respectively; the addition of unity is employed herein to reproduce the downward (incident) wave, which makes the PFR function closer to the conventional frequency response (FR) function.

The PFR function contains almost all the mechanics information below the sensors while eliminating the effect of the upper structure. A normalized semi-sine fictitious excitation with a unit amplitude and user-defined duration is then utilized to reconstruct the MSPDD result (as depicted in Figure 2b). This is the same as the result derived from a free-top PIT or lateral PIT [15,16] applied to a pile foundation that has no superstructure.

## 3. Multi-Task Learning Framework for Time-Series MSPDD Results

Benefiting from the TWD theory and the PFR function, the time-series MSPDD results, which are free from disturbances caused by environment, temperature and equipment, can reveal the reflections caused by the pile toe or pile defect below the sensors. Moreover, in post-processing, all parameters required for the reconstruction of the final MSPDD results (i.e., the duration of fictitious excitation, sampling frequency and time of the reconstructed signal) are user-defined and are not restricted by the hammer strike and testing device in practice. This means the MSPDD results obtained by various testing equipment can be utilized to build the sample set, which enables the data-driven identification of pile defects. Therefore, the time-series MSPDD results that are derived from original signals processed by TWD theory are utilized to conduct the pile damage detection, including multiple tasks: (1) number of defects below the sensors (**Task 1**); (2) type of defect (i.e., necking or expansion) closest to the sensors (**Task 2**); and (3) degree of the closest defect (**Task 3**). Once these tasks are resolved, the results can provide a detailed description of the actual pile quality and aid in classifying the pile quality.

### 3.1. Proposed Hybrid Convolutional and Recurrent Neural Network Framework

**Figure 3:**
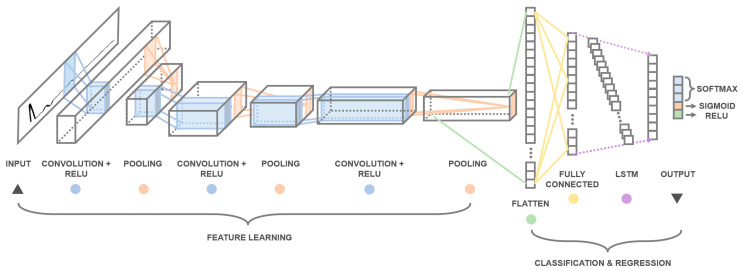
Architecture of hybrid 1D CNN and LSTM framework.

To conduct the multi-task pile damage detection, a hybrid one-dimensional (1D) convolutional and recurrent neural network is proposed herein to process the time-series MSPDD results. Figure 3 demonstrates the architecture of the proposed multi-task learning framework, which involves two major steps:

(1) One-dimensional convolutional neural network (CNN)

The time-series MSPDD results (whose length is equal to the user-defined number of sampling points) are input into the 1D convolutional neural network through the input layer. The 1DCNN is employed to automatically extract the features of the time-series signals. The proposed 1DCNN framework incorporates three pairs of convolution and pooling layers. The first convolution layer has 64 convolution kernels of size 5 with strides 1 and valid padding, while the second and third convolution layers have 128 and 256 convolution kernels, respectively, with the same size, strides and padding. The mathematical description of the convolution layer can be summarized as follows [40]:

(6) $$z_{i}^{l}=\sigma \left(\sum_{j}z_{j}^{l-1}\times w_{ij}^{l}+b_{i}^{l}\right)$$

where $z_{i}^{l}$ is the *i*th feature at layer *l*; $z_{j}^{l-1}$ is the *j*th feature at layer (*l* − 1); $w_{ij}^{l}$ denotes the kernel connecting the *j*th feature at layer (*l* − 1) and the *i*th feature at layer *l*; $b_{i}^{l}$ is the bias of the *i*th feature at layer *l*; and *σ*(·) denotes the activation function.

The outputs of the convolution layers are activated by Rectified Linear Unit (ReLU) and are then passed as input into their subsequent 1D max-pooling layers with a size of 4. The operation of the 1D max-pooling layer can be expressed as follows [40]:

(7) $$z_{p}^{l}=\begin{array}{c} \max\\ \forall i\in \Omega \end{array}z_{i}^{l-1}$$

where Ω denotes the pooling region.

(2) Long short-term memory (LSTM) network

The extracted features learned by 1DCNN are first flattened and reshaped, and then passed into the long short-term memory (LSTM) layers. The LSTM network belongs to the category of recurrent neural networks (RNNs) and can be utilized to learn the long-term dependencies of sequential inputs. The long-term memory of the network is achieved through the cooperation of three gate modules, i.e., forget gate (FG), update gate (UG) and output gate (OG). The operation of each gate module can be expressed as follows [41,42]:

(8) $$\mathrm{FG}:\;f_{t}=\sigma \left(W_{f}h_{t-1}+U_{f}x_{t}\right)$$

(9) $$\mathrm{UG}:\;\left\{\begin{array}{l} i_{t}=\sigma \left(W_{i}h_{t-1}+U_{i}x_{t}\right)\\ \tilde{C_{t}}=\tanh\left(W_{c}h_{t-1}+U_{c}x_{t}\right) \end{array}\right.$$

(10) $$\mathrm{OG}:\;\left\{\begin{array}{l} C_{t}=f_{t}C_{t-1}+i_{t}\tilde{C_{t}}\\ o_{t}=\sigma \left(W_{o}h_{t-1}+U_{o}x_{t}\right)\\ h_{t}=\tanh\left(C_{t}\right)o_{t} \end{array}\right.$$

where *W_f_* and *U_f_* are parameter matrices in the forget gate; *W_i_*, *W_c_* and *U_f_*, *U_c_* are parameter matrices in the update gate; *W_o_* and *U_o_* are parameter matrices in the output gate; and *x_t_*, *h_t_* and *C_t_* are the input, hidden state and cell state of the LSTM network at time step *t*, respectively.

### 3.2. Multi-Task Learning and Loss Function

The output (i.e., hidden state at the last time step) of the LSTM network is eventually utilized to resolve multiple tasks of pile damage detection as described above. Firstly, the Softmax activation is employed to classify pile damage conditions below the sensors into three categories: intact pile (**Class I**), pile with one defect (**Class II**) and pile with multiple (more than one) defects (**Class III**). The Softmax function can be expressed as follows:

(11) $$\mathrm{Softmax}\left(z_{i}\right)=p_{i}=\frac{\exp\left(z_{i}\right)}{\sum \exp\left(z_{i}\right)}$$

where the index *i* of the maximum probability *p_i_* corresponds to the classification of the pile damage conditions.

Secondly, the Sigmoid function is used to conduct the binary defect-type classification (necking or expansion) of the defect closest to sensors, i.e.,

(12) $$\mathrm{Sigmoid}\left(z_{i}\right)=\frac{1}{1+\exp\left(-z_{i}\right)}$$

Thirdly, the regression prediction of the degree of closest defect is obtained by ReLU activation, whose function is given as

(13) $$\mathrm{ReLU}\left(z_{i}\right)=\max\left(0,z_{i}\right)$$

So far, the output layer of size 5 neurons has been integrated to describe the actual pile quality below the sensors. Table 1 provides some examples of descriptions of output labels. Moreover, if the exact number or specific conditions of multiple defects need to be determined in some cases, it can also be achieved by continuing to lower the sensor position and repeating the MSPDD test. Multiple defects can then be detected one by one from top to bottom. However, in most cases, an RC pile with multiple defects will be directly evaluated as the worst class according to the Chinese Technical Code for Testing of Building Foundations Piles [43].

It is also noted that there exists a priority relationship between multiple tasks. Rationally, the binary classification (**Task 2**) and degree regression (**Task 3**) of the closest pile defect are only discussed when the pile is detected as defective; otherwise, the predictions of the latter two tasks are meaningless. Therefore, to clarify the sequence of multiple tasks and improve the accuracy of classification and regression as described in preceding sections, a loss function *J* is proposed to facilitate multi-task learning, i.e.,

(14) $$\left\{\begin{array}{l} J_{1}=-\sum_{i=1}^{3}y_{i}\cdot \log\left(p_{i}\right)\\ J_{2}=-\left[y_{4}\cdot \log\left(p_{4}\right)+\left(1-y_{4}\right)\cdot \log\left(1-p_{4}\right)\right]\\ J_{3}={\left(y_{5}-p_{5}\right)}^{2}\\ J=y_{1}\cdot J_{1}+\left(y_{2}+y_{3}\right)\cdot \left(J_{1}+J_{2}+J_{3}\right) \end{array}\right.$$

where *y_i_* and *p_i_* denote the *i*th element of the labeled and predicted output, respectively.

## 4. Sample Generation Based on Analytical Models

Wu et al. [35] demonstrated the excellent agreement of the reconstructed signals derived from the analytical solution and experimental results. Therefore, it is safe to say the MSPDD results are not sensitive to their sources (e.g., analytical solution, finite element analysis simulation or model test results). Given that the MSPDD results derived from actual integration test projects are insufficient at this point, the multi-channel signals collected by equally spaced sensors are simulated by an analytical solution herein to generate a sufficient number of samples and thus investigate the feasibility of employing the proposed hybrid neural network to conduct the multi-task learning.

The analytical solution to a pile with multiple defects embedded in layered soil when subjected to vertical excitation can be resolved by the scheme of acoustic impedance transfer mechanism (AITM) [11], where the acoustic impedance function *Z_i_* at the top of pile segment *i* (*i* ≥ 2) can be expressed as a function of *Z_i_*_−1_, i.e.,

(15) $$Z_{i}=\rho_{i}C_{i}\lambda_{i}\cdot \frac{\rho_{i}C_{i}\lambda_{i}\sin\left(\lambda_{i}/C_{i}\cdot l_{i}\right)-Z_{i-1}\cos\left(\lambda_{i}/C_{i}\cdot l_{i}\right)}{\rho_{i}C_{i}\lambda_{i}\cos\left(\lambda_{i}/C_{i}\cdot l_{i}\right)+Z_{i-1}\sin\left(\lambda_{i}/C_{i}\cdot l_{i}\right)}$$

where *ρ_i_*, C*_i_* and *l_i_* denote the mass density, one-dimensional elastic wave velocity and length of the pile segment *i*, respectively; *λ_i_* is a frequency-domain coefficient related to the resistance of soil around pile segment *i* and can be determined by existing dynamic soil models (e.g., Kelvin–Voigt model [4], Plane-strain model [44], Continuous media considering three-dimensional effects [11,12]).

**Figure 4:**
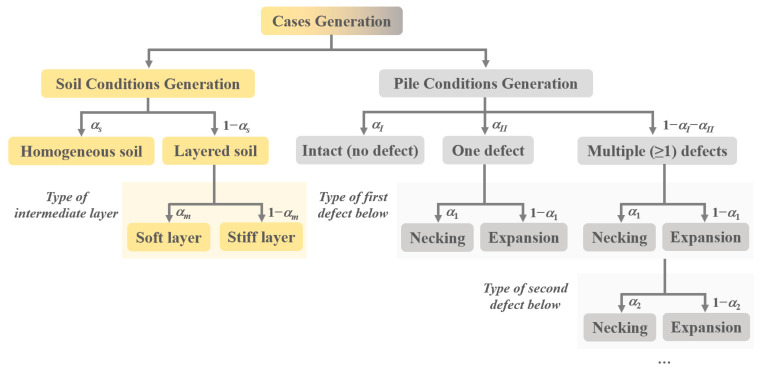
Flowchart of case generation.

Once the boundary conditions at the pile top and pile toe are determined, the dynamic responses along the pile shaft at various depths can be thoroughly solved. To obtain a better distribution of a sample set and facilitate multi-task learning, the analytical solution-based sample generation is artificially controlled. Figure 4 displays a flowchart of the generation procedure of various cases for analytical analysis, where *α_I_* and *α_II_* denote the occurrence probabilities of various pile conditions; *α*_1_ and *α*_2_ are the occurrence probabilities of the type of the first and second defects below the sensors, respectively; and *α_s_* and *α_m_* are the occurrence probabilities of various soil conditions and type of the intermediate layer, respectively.

**Figure 5:**
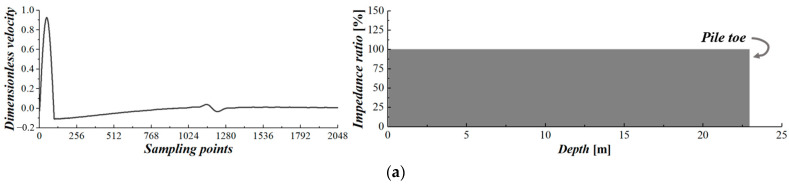

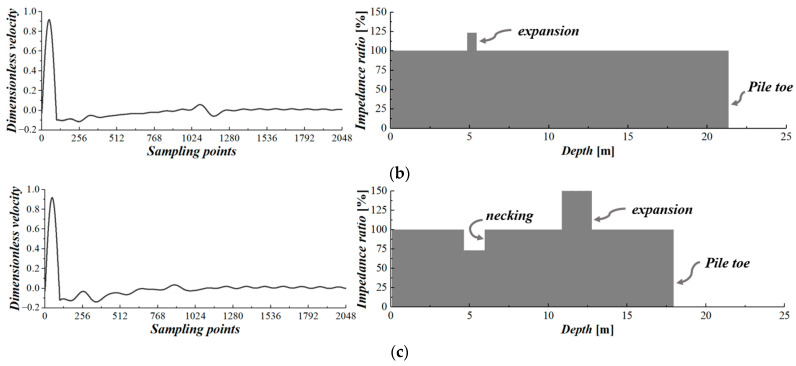
MSPDD results derived from multi-channel signals simulated by analytical solutions: (**a**) intact pile; (**b**) pile with one defect; (**c**) pile with multiple (more than one) defects.

The analytical results subject to the generated random cases are added with additive white Gaussian noise (AWGN) to increase the randomness of the raw data. The noise-introduced analytical results are then utilized to simulate the time-series signals collected by multiple equally spaced sensors of the MSPDD test. After the post-processing procedure described in Section 2.2, the simulated multi-channel signals are finally processed into a sample set of the MSPDD results. Figure 5 displays several MSPDD results derived from multi-channel signals simulated by analytical solutions subjected to specific cases, where the reconstructed signals can eliminate the effect of superstructures and thus highlight the reflections below the sensors. It is also noted that the MSPDD results have already been normalized by introducing the generalized incident wave as clarified in Section 2.2.

## 5. Results and Discussion

An analytical solution-based sample set is utilized herein to verify the feasibility of employing the proposed hybrid convolutional and recurrent neural network to conduct multi-task pile damage detection. The sample set is randomly split into training, validation and testing sets (60/20/20%); the parameters of the hybrid neural network are tuned by the training and validation sets, and the performance of the multi-task pile damage detection is evaluated by the testing set. Moreover, the early stopping technique is employed to reduce overfitting without compromising on model accuracy.

**Figure 6:**
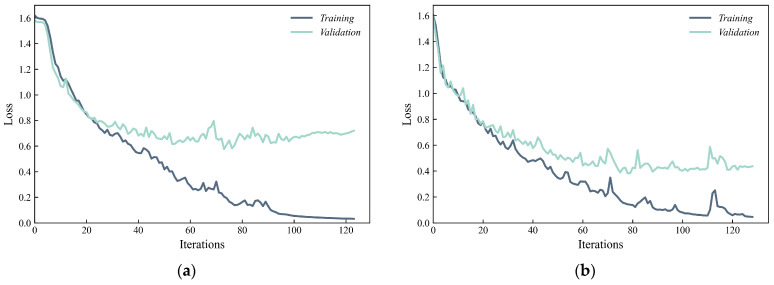

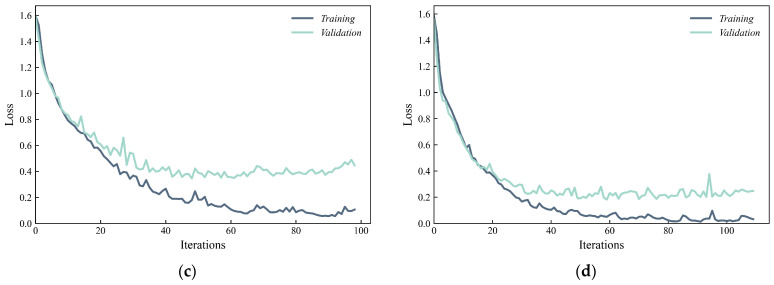
Iterative learning procedure of loss function considering number of samples: (**a**) 1000; (**b**) 2000; (**c**) 4000; (**d**) 8000 (loss function is expressed in Equation (14)).

Given that the collection of MSPDD results derived from actual projects is time-consuming and labor-intensive, it is reasonable to take the size of the sample set into consideration. Figure 6a–d demonstrate the iterative learning procedure of the loss function considering sample numbers equal to 1000, 2000, 4000 and 8000. As expected, as the number of samples increases, the loss function drops faster and the validation loss derived from the validation set converges to a smaller value, which corresponds to better learning efficiency. Taking advantage of the early stopping technique, the iteration stops when the validation loss has a tendency to increase in later iterations, which can effectively suppress overfitting and strengthen the generalization ability of the model, especially for a small sample set (e.g., Figure 6a).

**Figure 7:**
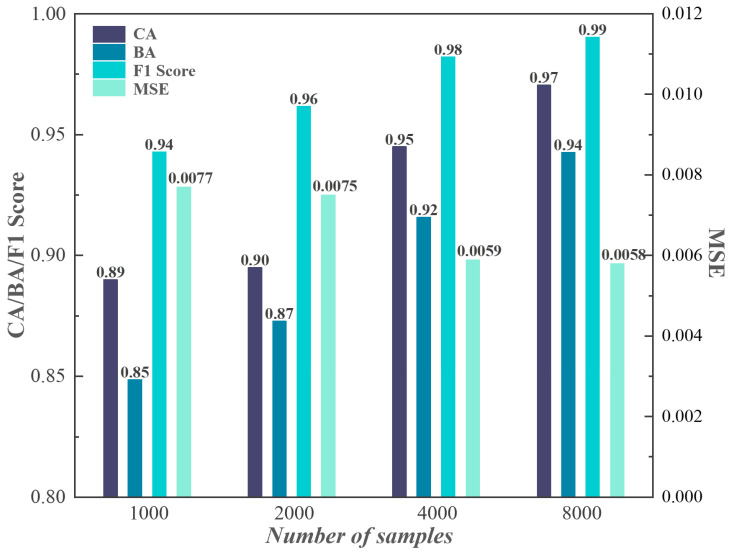
Performance of hybrid neural network on multi-task pile damage detection.

Figure 7 displays the overall evaluation of the proposed hybrid neural network in multi-task pile damage detection. Four accuracy metrics are introduced to evaluate the hybrid model performance on specific pile damage detection tasks: (1) Categorical Accuracy (CA) for multi-classification of pile conditions (**Task 1**); (2) Binary Accuracy (BA) for type classification of the closest pile damage (**Task 2**); (3) Mean Squared Error (MSE) accuracy for degree regression of the closest pile damage (**Task 3**); (4) F1 Score for binary classification (pile is defective or not), i.e., recognition of **Class I**, which is a sub-task of **Task 1** (denoted as **Task 1-I**). The formula for the F1 Score can be expressed as follows:

(16) $$F_{1}=2\cdot \frac{precision\cdot recall}{precision+recall}$$

Consistent with the tendency of the loss function, CA, BA and F1 Score values increase and the MSE value decreases as the sample size increases. Fortunately, benefiting from the proposed multi-sensor detection method as well as the multi-task learning framework, the accuracy metrics for small samples (e.g., 1000 in this case) are also satisfactory. Comparing CA, BA and F1 Score values, it is worth noting that the recognition accuracy of **Class I** is better than the Categorical Accuracy of multi-class (**Class I, II and III**), and both are better than the defect-type classification accuracy. This is due to the sequential relation between multiple tasks, where **Task 2** is conducted based on the sub-task of **Task 1** (i.e., **Task 1-I**), while **Task 3** is conducted on the basis of **Task 2**. Although errors from the previous task are passed on to its subsequent task, this scheme also follows the priority of the detection order in an actual project. For the purpose of only identifying whether there are defects below the sensors (i.e., **Task 1-I**), even a small sample set (e.g., 1000 in this case) can obtain an excellent recognition accuracy (F1 Score = 0.94) based on the hybrid model. Furthermore, if the number and type of defects need to be determined in some cases, a larger sample size is required (≥4000 in this case) to obtain satisfactory CA (≥0.95) and BA (≥0.92) values.

**Figure 8:**
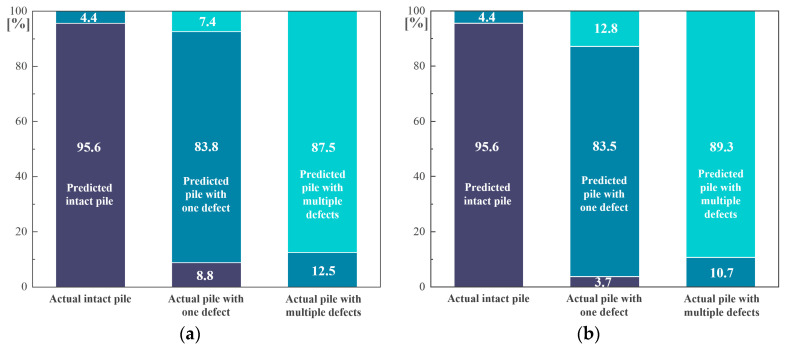

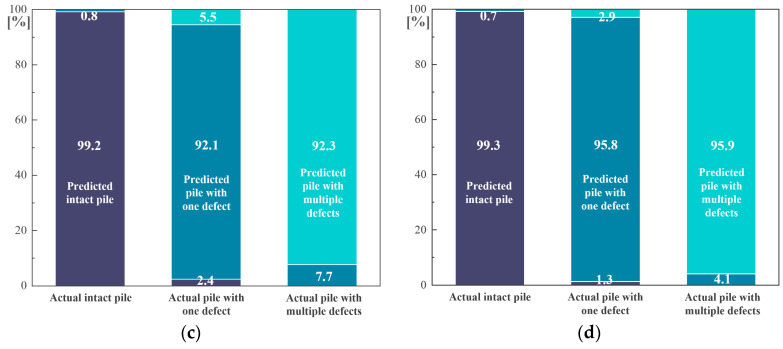
Performance of hybrid neural network in **Task 1** considering numbers of samples equal to (**a**) 1000; (**b**) 2000; (**c**) 4000; (**d**) 8000.

Figure 8 further investigates the performance of the proposed model on **Task 1**. As indicated by the F1 Score in Figure 7 and Figure 8a–d, the recognition precision and recall of the intact pile condition (i.e., **Class I**) are satisfactory regardless of the sample size. Only a small proportion of piles with one defect (**Class II**) are misidentified as intact piles (**Class I**), and the proportion continues to decrease to 1.3% as the sample size increases to 8000. In engineering practice, it is quite important to accurately distinguish between intact piles and defective piles (with one or multiple defects), thus the importance of **Task 1-I** even exceeds **Task 1** itself. From this perspective, the hybrid model is competent for the basic task of pile damage detection, and it also performs well in small sample cases. As a comparison, the confusion between the recognition of piles with only one defect (**Class II**) and piles with multiple defects (**Class III**) is more pronounced in this case. However, as the number of samples increases, this confusion also diminishes to a satisfactory level, where the precision/recall rates of **Class II** and **Class III** both exceed 95% when the sample size reaches 8000.

**Figure 9:**
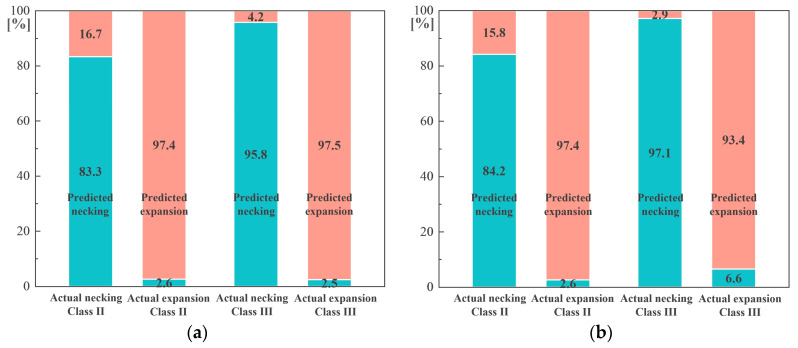

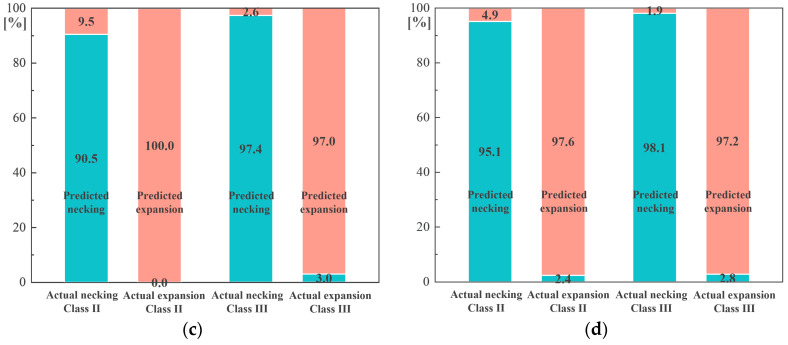
Performance of hybrid neural network in **Task 2** considering numbers of samples equal to (**a**) 1000; (**b**) 2000; (**c**) 4000; (**d**) 8000.

The performance of the hybrid neural network in **Task 2** is demonstrated in Figure 9a–d. Similarly, the recognition accuracy of defect type increases as the sample size becomes larger. If we take the overall recognition accuracy over 95% as the standard, it is appropriate to construct a sample set of more than 4000 samples to conduct this task. It is also noted that the type recognition of the defect of **Class III** performs better than that of **Class II**, while the accuracy of expansion recognition is also higher than that of necking recognition. The reason for this phenomenon may be that the reflection caused by a necking defect is confused with the pile toe reflection for friction piles, especially when there is only one defect between the sensors and the pile toe.

**Figure 10:**
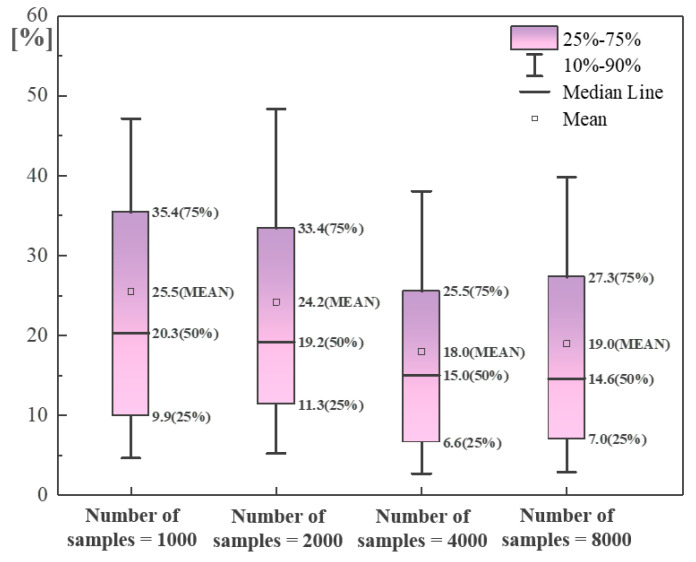
Performance of hybrid neural network in **Task 3**.

Figure 10 displays the performance of the hybrid neural network in **Task 3**, where the percentage deviation of the predicted defect degree is demonstrated by the box plot. The deviations corresponding to the medians derived from different sample sizes are basically lower than 20%. As the sample size increases, the mean/median values decrease while the box/whisker range almost remains the same, which means a larger sample size is not beneficial for reducing the variance. This is because the regression of the defect degree is inevitably affected by previous tasks (i.e., residual term of the loss function), and the predicted value output from the hybrid neural network can only be used as a reference value; however, this is sufficient for the detailed description of the pile quality since the number and type of defects have been determined.

## 6. Conclusions

(1) A novel multi-sensor pile damage detection (MSPDD) method is proposed in this paper to evaluate the integrity of onshore and offshore pile foundations, while the traveling wave decomposition (TWD) theory is utilized to implement the post-processing for a series of signals collected by multiple sensors. The reconstructed MSPDD results are then utilized to conduct automatic pile damage detection with multiple tasks.
(2) A hybrid one-dimensional (1D) convolutional and recurrent neural network is developed for the time-series MSPDD results, and a loss function is proposed to clarify the sequence between multiple tasks and therefore facilitate multi-task learning. Then, an analytical solution-based sample set is utilized to verify the feasibility of employing the hybrid model to conduct multi-task pile damage detection.
(3) Benefiting from the proposed multi-sensor detection method as well as the multi-task learning framework, the accuracy metrics derived from different sample sizes are satisfactory. For the purpose of only identifying whether there are defects below the sensors (i.e., **Task 1-I**), even a small sample set (e.g., 1000 in this case) can obtain an excellent recognition accuracy (F1 Score = 0.94) based on the hybrid model. If the number and type of defects need to be determined in some cases, a larger sample size is required (e.g., ≥4000 in this case) to obtain satisfactory CA (≥0.95) and BA (≥0.92) values.
(4) The recognition accuracy of defect type increases as the sample size becomes larger. It is appropriate to construct a sample set of more than 4000 samples to reach 95% overall recognition accuracy, where the accuracy of expansion recognition is higher than that of necking recognition. The deviations of predicted defect degrees corresponding to the medians are basically lower than 20%, which can provide a more detailed description of the pile quality together with the number and type of defects.

## Figures and Tables

**Table 1:** Some examples of descriptions of output labels.

Labels of Outputs	Description (Below Sensors)
$\left[\begin{array}{ccccc} 1 & 0 & 0 & 0 & 0 \end{array}\right]$	There is no defect.
$\left[\begin{array}{ccccc} 0 & 1 & 0 & 0 & 0.31 \end{array}\right]$	There is one defect, whose acoustic impedance value is 31% lower than that of the intact segment.
$\left[\begin{array}{ccccc} 0 & 0 & 1 & 1 & 0.16 \end{array}\right]$	There are multiple defects, and the acoustic impedance value of the closest defect is 16% higher than that of the intact segment.

## Data Availability

Data are contained within the article.
